# Microcapillary-assisted acoustoelectric platform for selective single-cell isolation and in situ transfection

**DOI:** 10.1038/s41378-026-01490-z

**Published:** 2026-10-19

**Authors:** Xuefeng Xu, Ziqiao Zhou, Guangyang Li, Jie Liang, Jianhui Wu, Jiaqi Lu, Fang Shen, Shengjun Wu, Zhi Ruan, Shurong Dong, Jikui Luo, Zhen Cao

**Affiliations:** 1 College of Information Science and Electronic Engineering, Zhejiang University, Hangzhou, 310027 China; 2 International Research Center for Information Science and Electronic Engineering, Zhejiang University, Haining, 314400 China; 3 Department of Clinical Laboratory, Shaoxing Second Hospital, Shaoxing, China; 4 Department of Clinical Laboratory, Sir Run Run Shaw Hospital, Zhejiang University School of Medicine, Hangzhou, 310016 China; 5 Zhejiang Provincial Engineering Research Center of Innovative Instruments for Precise Pathogen Detection, Hangzhou, 310016 China; 6 Zhejiang Key Laboratory of Advanced Micro-Nano Transducers Technology, Zhejiang University, Shaoxing, 312000 China

**Keywords:** Electrical and electronic engineering, Chemistry

## Abstract

Single-cell transfection technologies hold tremendous promise for precision cell and immunotherapies. However, monolithic integration of cell isolation with targeted intracellular delivery remains technically challenging, especially within complex biological matrices. Herein, we present a microcapillary-assisted acoustoelectric single-cell selective isolation and sonoporative transfection platform (μC-ASSIST), which seamlessly sequences microcapillary-assisted dielectrophoresis (μC-DEP) for deterministic single-cell trapping with high-frequency surface acoustic waves (SAWs) for transient membrane poration within a unified workflow. We demonstrate the label-free, high-efficiency extraction of human non-small cell lung cancer (A549) cells from a complex white blood cell (WBC) background with a capture efficiency of 95%. Furthermore, we validate the highly efficient, contact-free intracellular delivery of diverse cargoes ranging from small dyes (PI) to functional small interfering RNA (siRNA) and large plasmid DNA (mRFP-C3), maintaining a high cell viability of 83.7% alongside a 78.1% transfection efficiency. Crucially, the targeted delivery of siBcl-xL significantly suppressed target mRNA expression and profoundly inhibited tumor cell motility. By circumventing off-target effects and cumbersome manual handling, the μC-ASSIST platform establishes a highly versatile, streamlined paradigm for precision single-cell engineering, holding immense potential for advancing functional genomics and next-generation gene therapies.

**Figure:**
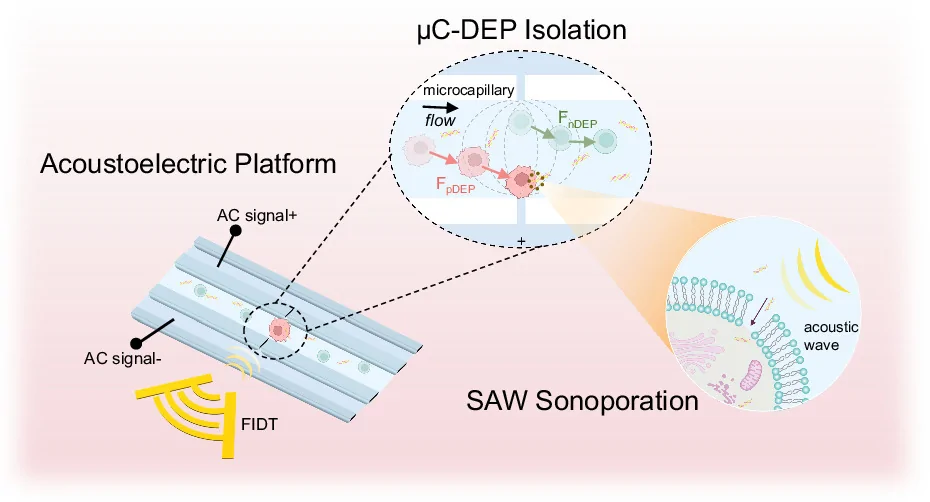

## Introduction

Metastasis, the primary driver of cancer-related mortality, arises from the dissemination of tumor cells from the primary site into the peripheral bloodstream, leading to the colonization of distant microenvironments^1^. Despite the increasing knowledge on the extravasation process and concerted efforts devoted to the development of cancer therapeutics, the underlying cellular and molecular mechanisms governing this multistep cascade remain elusive^2^. Understanding the complex signaling pathways and their effectors that regulate cancer extravasation can profoundly benefit from single-cell analysis, which interrogates cellular heterogeneity to reveal diverse functional states and varying drug responses among cellular phenotypes^3,4^. Obviously, there is a great incentive for experimental platforms that enable fast and robust isolation of target tumor cells and downstream cellular engineering and analysis. A plethora of techniques have been adapted to perform cell assays individually for various parametric information, including mass spectrometry and flow cytometry^5,6^, which allow high-sensitivity and multiplex measurement. Still, they inherently suffer from critical drawbacks. First, these bulk-processing technologies require substantial starting sample volumes. Minute sample losses that are common and negligible at the population level may be devastating when analyzing rare single cells. Second, labor-intensive manual pretreatment steps compromise the temporal resolution necessary to capture transient single-cell dynamics. Third, fluorescence-based methodologies are frequently constrained by specimen photodamage and fluorophore photobleaching, hampering prolonged downstream functional analyses.

Recently, microfluidics has emerged as a powerful paradigm for the isolation and manipulation of single cells with improved efficiency and recovery rate^7^. By handling and processing fluidics at micrometer or even nanometer scales, these miniaturized devices provide great advantages including sample economy, simplified operation, cost-effectiveness, rapid analysis time, and portability^8^. Among microchip-based platforms, microchamber chips were commonly utilized to physically compartmentalize cells for culture and reactions without cross-contamination^9^. However, these architectures typically rely on complex multilayer fluidics and integrated micro-valves, resulting in high operational complexity and restricted throughput. Alternatively, droplet-based microfluidics facilitates the parallel, high-throughput profiling of encapsulated single cells^10–12^. Yet, droplet-based approaches generally require high initial cell concentrations and lack the capability for the deterministic, sequential probing of specifically targeted individual droplets. Other active techniques have been explored for single-cell manipulation and isolation, including electrical^13,14^, acoustic^15–17^ and magnetic forces^18,19^. Despite progress in isolation flexibility, these standalone platforms typically perform single-function analyses on intact cells, severely restricting their accessibility to study intracellular variables or generating genetically engineered single cells.

For the advancement of precision cancer cell and gene therapies, the efficient intracellular delivery of target cargoes across the plasma membrane remains a critical bottleneck in gene-editing methodologies^20–22^. Among various delivery strategies, electroporation is well-established and predominantly employed due to its simple operation and high reproducibility^23,24^. By tuning the electric field intensity and duration, hydrophilic nanopores can be created across the cell membrane for ex vivo delivery with reduced safety concerns. Nonetheless, the usage of strong fields leads to substantial cell damage or unintended DNA double-strand breaks. To address these issues, some mechanoporation methods are proposed, including microneedles^25,26^ and cell squeezing^27^. More recently, acoustofluidic sonoporation driven by acoustic cavitation has proven effective for membrane permeabilization and intracellular delivery^28–30^. However, the vast majority of sonoporation studies operate on bulk cell populations. The capability to monolithically and seamlessly integrate the highly specific isolation and in situ poration of target single cells remains an unmet, acute need for clinical oncology and personalized cell therapies.

To bridge this technological gap, we introduce a microcapillary-assisted acoustoelectric single-cell selective isolation and sonoporative transfection chip (μC-ASSIST) that integrates target-specific capture and on-site molecular delivery within a streamlined, automated workflow. By harnessing enhanced electric field gradients at the microcapillary orifices, we achieve high-purity dielectrophoretic (DEP) capture of single target tumor cells from complex, heterogeneous sample matrices. To ensure cellular safety, we established a biocompatible operating regime by systematically optimizing excitation frequencies and voltage amplitudes, effectively circumventing electrical toxicity. For the subsequent intracellular delivery, high-frequency surface acoustic waves (SAWs) were employed to induce transient membrane poration, achieving an optimal equilibrium between high delivery efficiency and the preservation of cellular integrity. The platform’s versatility is validated through the successful cytosolic and nuclear delivery of diverse molecular cargoes, including small-molecule dyes, functional plasmids, and small interfering RNA (siRNA). Notably, the targeted delivery of siBcl-xL successfully knocked down anti-apoptotic gene expression and profoundly suppressed cancer cell motility, demonstrating the platform’s capacity for potent phenotypic modulation. The μC-ASSIST platform provides a robust, scalable, and versatile solution for precision single-cell surgery, offering significant potential for applications in functional genomics and personalized oncology.

## Results and discussion

### Concept and working principle of μC-ASSIST chip

**Fig. 1:**
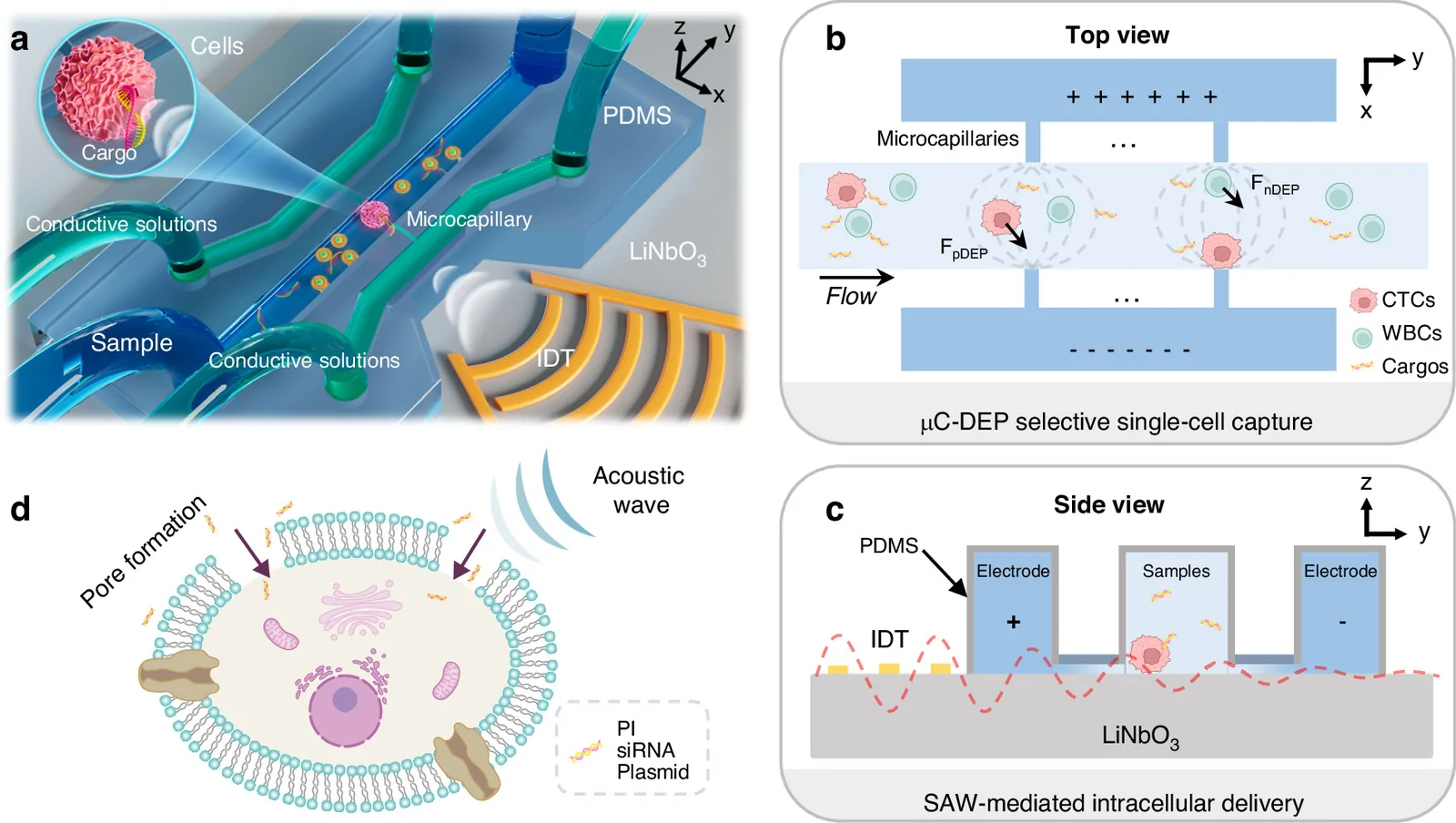
**Integrated μC-ASSIST chip for selective single‑cell capture and biomolecule delivery.** **a** Schematic of the μC-ASSIST chip comprising a sample channel, two liquid‑electrode side channels, and FIDT on a LiNbO₃ substrate. **b** Schematic diagram of the single-cell selective capture using μC-DEP. Frequency‑tuned DEP enables tumor cell trapping at microcapillary orifices (pDEP) while WBCs undergo nDEP and are flushed away. **c** Schematic of acoustically mediated intracellular delivery. The tSAW induces transient membrane poration, permitting uptake of exogenous biomolecules. **d** In situ delivery of PI, siRNA, and plasmid DNA into a single tumor cell by acoustic wave

Figure 1a shows the schematic of the developed μC-ASSIST chip, which consists of a piezoelectric lithium niobate (LiNbO_3_) substrate patterned with a focused interdigital transducer (FIDT) and a polydimethylsiloxane (PDMS) slab embedded with fluidic channels. The main sample channel (blue) is flanked by two side channels (green) interconnected via microcapillaries. These side channels and microcapillaries are filled with a high-conductivity solution to function as 3D liquid electrodes, efficiently coupling the electric fields into the main channel. This configuration significantly simplifies the fabrication process by obviating the requirement for complex surface microelectrodes, while generating a highly non-uniform electric field distribution. Within this non-uniform gradient, cells exhibited frequency-dependent DEP responses dictated by their sizes and dielectric properties. The magnitude and direction of DEP forces are governed by the square of the electric field gradient (∇|*E*|²) and the Clausius–Mossotti (CM) factor. The real part of the CM factor (${{{\rm{R}}}}{{{\rm{e}}}}[{\widetilde{f}}_{{{{\rm{CM}}}}}]$) directly determines whether the cell experiences positive DEP (pDEP, ${{{\rm{R}}}}{{{\rm{e}}}}\left[{\widetilde{f}}_{{{{\rm{CM}}}}}\right] > 0$) or negative DEP (nDEP, ${{{\rm{R}}}}{{{\rm{e}}}}\left[{\widetilde{f}}_{{{{\rm{CM}}}}}\right] < 0$)^31^. Detailed theoretical derivations are provided in Section S1. By optimizing the applied frequency and the medium conductivity, target cells such as tumor cells can be selectively and effectively trapped at the microcapillary orifices by pDEP, where the field gradient is significantly intensified by the reduced curvature of round openings (Fig. 1b). Moreover, unlike conventional planar electrodes where the DEP forces decay exponentially away from the metal surface, these 3D capillary electrodes exert a volumetric force spanning the entire chamber height. In contrast, non-target cells, including WBCs, experience nDEP and are repelled toward regions with lower field gradients, allowing their continuous removal via bulk flow to the outlet. Consequently, this mechanism enables the highly selective capture of individual cells from a complex heterogeneous mixture.

Following selective capture of tumor cells by the microcapillary‑assisted DEP (μC-DEP), a traveling surface acoustic wave (tSAW) is applied via the FIDT to induce transient membrane poration, enabling the efficient uptake of exogenous biomolecules into the cells from the surrounding medium (Fig. 1c). In contrast to existing sonoporation strategies that necessitate cell adherence or complex biochemical tethering^29,32^, our platform achieves the label-free, selective immobilization of individual suspension cells. During SAW exposure, the immobilized cell experiences the acoustic radiation force and the viscous drag associated with acoustic streaming. However, the DEP field remains continuously applied during acoustic treatment, and the near-field DEP force is substantially larger than the acoustic-induced displacing forces under the optimized conditions. Together with the geometric confinement provided by the microcapillary opening, the maintained DEP confinement prevents substantial cell displacement during SAW exposure. Although cell occupation redistributes the local electric field and attenuates ∇|*E*|² near the microcapillary opening, the combined effects of electrical trapping and physical confinement are sufficient to preserve stable cell positioning during acoustic treatment. As theoretically detailed in Section S2 and Fig. S1, this firm immobilization ensures robust acoustic energy coupling into the lipid bilayer, creating temporary hydrophilic pores. Consequently, it facilitates the contact-free delivery of diverse cargoes, including small molecules, nucleic acids, and proteins, into the target captured tumor cells while maintaining high long-term viability (Fig. 1d). Following on-chip processing, the transfected cells can be easily retrieved for downstream gene editing or therapeutic profiling. As a proof of concept, we performed in situ transfection of siRNA (siBcl-xL) to interrogate the function of the Bcl-xL gene, which encodes a key anti-apoptotic protein. Given that Bcl-xL expression is intrinsically linked to tumor cell survival, invasion, and metastasis, its targeted silencing serves as a robust paradigm for gene-based cancer therapy^33,34^. The phenotypic impact of this silencing effect was quantitatively validated via a wound healing assay^35^. The observed inhibition of cell migration confirms that our device not only executes highly efficient biomolecular delivery but also triggers definitive, therapeutically relevant downstream cellular responses. Ultimately, by integrating selective capture and transfection into a seamless workflow, this platform offers a versatile and scalable tool for single-cell analysis and combinatorial therapeutic development.

### Regulation of electric field gradients via microcapillary arrays

**Fig. 2:**
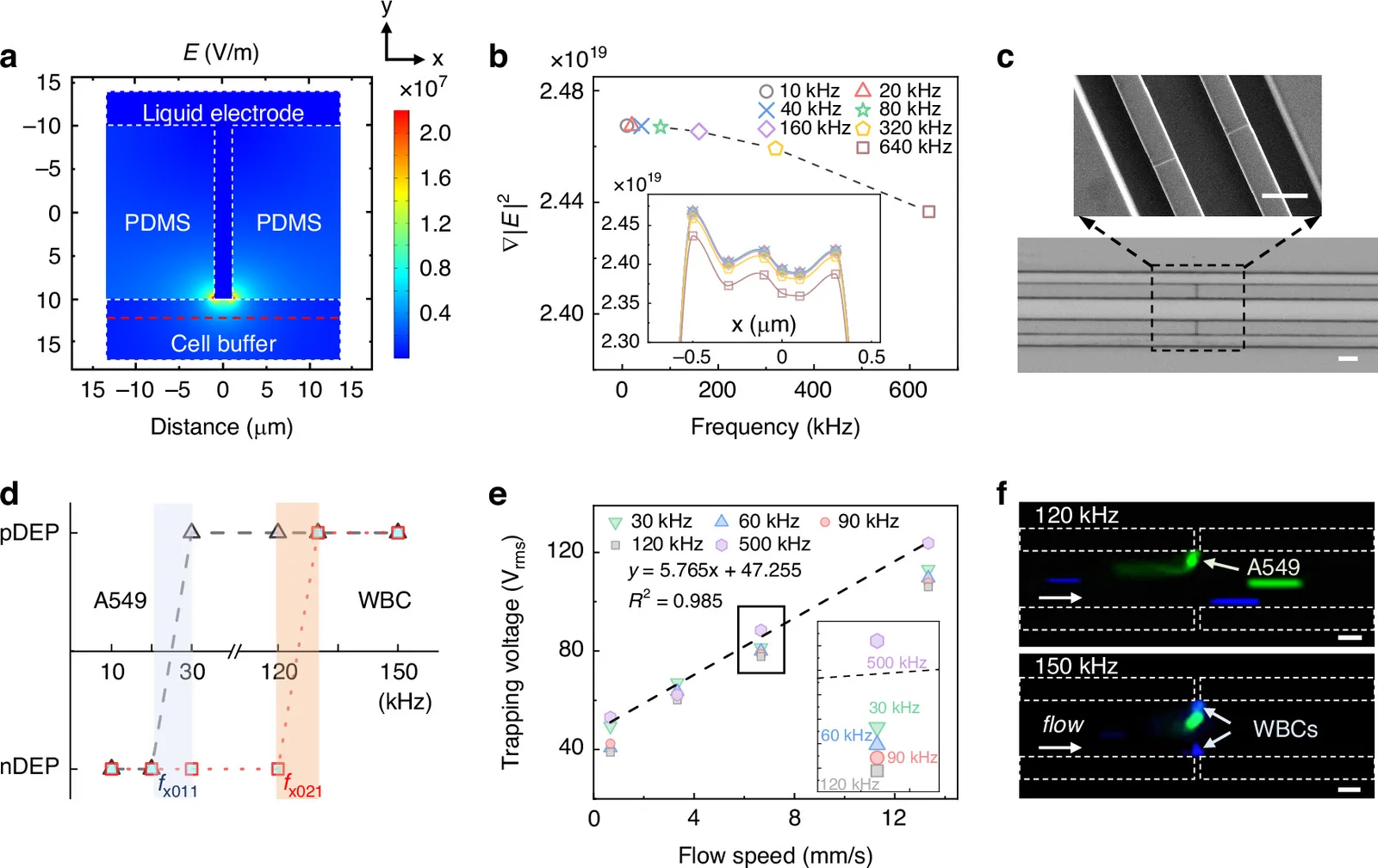
**μC-DEP for selective single-cell capture.** **a** Simulated electric field distribution near a single microcapillary in the *x*–*y* plane at *f* = 160 kHz, *V*_rms_ = 21.2 V. **b** Frequency-dependent maximum ∇|*E*|² near the microcapillary opening (*y* = 12 μm, red line in **a**) at a fixed *V*_rms_ = 21.2 V. **c** The optical and SEM images of the channel and a pair of microcapillaries. Scale bars: 40 μm. **d** Experimentally determined crossover frequencies for the nDEP-to-pDEP transition in A549 cells and WBCs (*n* > 10 for each cell type). **e** Minimum voltage required for single-cell trapping (A549) as a function of flow speed at different frequencies. **f** Representative fluorescence images of A549 cell capture at 120 kHz and 71 *V*_rms_, showing A549 cells (green) trapped at microcapillaries while WBCs (blue) flow through. At 150 kHz, both cell types are trapped. Scale bars: 20 μm

Conventional DEP techniques for single-cell capture are frequently constrained by a lack of deterministic capture sites, diffuse electric field gradients, and insufficient capture forces^36,37^. To address these limitations, we integrated a microcapillary structure to precisely modulate and confine the electric field for single-cell capture using DEP. To elucidate the underlying mechanism, the electric field distribution of a single microcapillary (2 μm × 2 μm × 20 μm) within a PDMS partition was computationally evaluated. The simulation modeled the side channels and a microcapillary filled with a conductive electrolyte, interfaced with the sample medium in the main channel. As depicted in the surface plots (Figs. 2a and S2a), under a 160 kHz and 21.2 *V*_rms_ excitation, the electric field is highly intensified around the capillary orifice. The local field strength peaks at 10^7^ V/m at the corner, with a ∇|*E*|² exceeding 10^19^ V²/m³. A comprehensive comparison with existing platforms (Table S1) reveals that the ∇|*E*|² generated by our configuration is 3–4 orders of magnitude higher than that of traditional insulator-based DEP (iDEP) devices, and 1–2 orders greater than that of planar electrode-based DEP platforms. This robust enhancement effectively circumvents the chronic flaw of conventional iDEP systems, which typically yield weak gradients despite demanding high actuation voltages^38,39^. The mechanism fundamentally relies on the electro-fluidic coupling between the microcapillary and the main channel; the severely restricted cross-section structurally distorts and compresses the electric field lines. This geometric constriction generates a highly localized, non-uniform field profile. Consequently, cells experiencing pDEP are deterministically steered toward and securely anchored at these ∇|*E*|² maxima, achieving precise and high-fidelity single-cell positioning.

To further elucidate the physical mechanism underlying this gradient enhancement and its transition during cell capture, we developed equivalent circuit models for the fluidic device under both unloaded and cell-loaded states (Fig. S2c, d). In the unloaded state, the electrolyte-filled microcapillary functions as a resistive element (${R}_{{{{\rm{\mu }}}}{{{\rm{C}}}}}$), while the insulating PDMS partition acts as a capacitor (${C}_{{{{\rm{PDMS}}}}}$). The macroscopic electric field distribution is fundamentally governed by the interplay between the resistive conduction through the microcapillary and the capacitive coupling across the partition. Based on this analogy, we evaluated the frequency-dependent response of the electric field distribution. Figure 2b plots the maximum ∇|*E*|² near the microcapillary orifice from 10 to 640 kHz at a fixed 21.2 *V*_rms_ excitation. Notably, ∇|*E*|² exhibits a decline as the frequency increases, particularly above 160 kHz. This attenuation is attributed to the frequency-dependent impedance of the PDMS partition (${Z}_{{{{\rm{PDMS}}}}}\propto \frac{1}{{{{\rm{j}}}}w{C}_{{{{\rm{PDMS}}}}}}$). At lower frequencies, the high impedance of the PDMS forces the electric current to flow predominantly through the microcapillary, ensuring maximal spatial field confinement. Conversely, as the frequency increases, the declining capacitive reactance causes capacitive coupling to dominate. Consequently, a larger fraction of electric field lines penetrates the dielectric partition instead of constricting through the microcapillary, thereby diminishing the field gradient in the main channel. Considering the need for strong localized electric-field gradients while limiting electrochemical polarization and unintended electroporation, the 10–160 kHz range was identified as a favorable operating regime. Within this optimal range, the localized and pronounced electric field gradients around the capillary facilitate robust dielectrophoretic trapping at relatively low actuation voltages. Once a cell occupies the microcapillary opening, the device transitions to a cell-loaded electrical state (Fig. S2d). The membrane, cytoplasm, and residual fluid-filled gap introduce additional impedance components that perturb the preferential current conduction through the microcapillary and reshape the electric field distribution. As shown in Fig. S2a, b, ∇|*E*|² along the axial sampling path decreases by more than one order of magnitude within the first 15 μm extending from the opening into the main channel, thereby weakening the DEP attraction acting on subsequently approaching cells. A secondary local maximum appears near the cell-medium interface, but this region is located closer to the center of the main channel, where the higher flow velocity and hydrodynamic drag are unfavorable for stable secondary trapping. Together, these effects reduce the likelihood of multiple cells being captured at the same microcapillary and contribute to a self-limiting single-cell trapping mechanism.

Building upon the equivalent circuit model, the electric field distribution is also influenced by the ${R}_{{{{\rm{\mu }}}}{{{\rm{C}}}}}$, a parameter intrinsically linked to the microcapillary geometry. To systematically evaluate these geometric effects on tumor cell trapping efficacy, the electric field gradients were simulated at a representative frequency of 100 kHz (Fig. S3). Varying the capillary length from 10 to 200 μm resulted in only minor fluctuations of the maximum ∇|*E*|² within the same order of magnitude, primarily due to marginal changes in the curvature at the microcapillary orifice. Conversely, increasing the capillary width from 1 to 8 μm induced a pronounced reduction in the maximum field gradient. This observation confirms that a narrower cross-section more strictly constrains the electric flux, thereby forcing stronger distortion and concentration of the electric field lines. Balancing these simulation insights with practical fabrication feasibility, an optimal capillary width of 2 μm was determined for this study. Utilizing these optimized design parameters, the microfluidic device was fabricated using CMOS-compatible processes. Representative optical and scanning electron microscopy (SEM) images of the fabricated chip are displayed in Fig. 2c, with comprehensive fabrication procedures detailed in the “Materials and methods” section and Fig. S4.

### Microcapillary-assisted DEP for selective single-cell capture

The efficacy of the selective capture relies on the intrinsic dielectric disparity between the target tumor cells and the background blood cell populations. Using A549 cells as a model, we first theoretically derived the optimal separation parameters by computing the frequency-dependent ${{{\rm{R}}}}{{{\rm{e}}}}\left[{\widetilde{f}}_{{{{\rm{CM}}}}}\right]$ based on established cell properties (Table S2). Considering the strong dependence of the CM factor on the conductivity of the suspending medium, we evaluated the frequency-dependent dielectric responses of A549 cells and WBCs at medium conductivities of 10, 100, and 1000 μS/cm (Fig. S5). As illustrated in Fig. S5, increasing the medium conductivity progressively shifts the crossover frequencies of both cell types toward higher frequencies while reducing the magnitude of the positive CM factor. Conversely, at low conductivity, the crossover frequencies shift into a very low-frequency regime, where electrode polarization, electrochemical effects, and membrane charging become increasingly unfavorable for stable cell manipulation. Therefore, a medium conductivity of 100 μS/cm was selected as a practical compromise between dielectric selectivity, DEP response, and cellular compatibility. At this conductivity, while the ${{{\rm{R}}}}{{{\rm{e}}}}\left[{\widetilde{f}}_{{{{\rm{CM}}}}}\right]$ of both cell types converge at high frequencies, a significant divergence emerges in the lower frequency regime (<200 kHz). We define the crossover frequency (${f}_{{{{\rm{x}}}}0}$) as the threshold at which cells transition from an nDEP to a pDEP response. Specifically, A549 cells exhibit a first crossover frequency (${f}_{{{{\rm{x}}}}011}$) of approximately 30 kHz, whereas that of WBCs is markedly higher at ~120 kHz. This spectral gap is experimentally validated in Fig. 2d, demonstrating measured crossover frequency ranges of 20–30 kHz for A549 cells and 120–130 kHz for WBCs. This distinct frequency-dependent behavior establishes an optimal operational window (30–120 kHz) for differential DEP sorting. Crucially, this biological frequency window strategically aligns with the optimal physical performance of our device. As simulated earlier (Fig. 2b), lower frequencies induce stronger ∇|*E*|² near the microcapillary orifices. To evaluate how this spatial field concentration translates to trapping robustness against hydrodynamic drag, we experimentally determined the minimum voltage required to sustain single-cell immobilization across varying flow rates (Fig. 2e). Consistent with Stokes’ law, higher flow rates demanded a linear increase in trapping voltage to counteract the enhanced hydrodynamic drag. Interestingly, the frequency profoundly modulated this voltage requirement. At elevated frequencies (e.g., 500 kHz), capacitive leakage across the microfluidic insulating structures severely attenuated the ∇|*E*|², thereby forcing a sharp increase in the requisite trapping voltage. In contrast, at lower frequencies, the sensitivity of the trapping voltage to the flow rate was significantly dampened, evidenced by the reduced slopes of the response curves. Although the ${{{\rm{R}}}}{{{\rm{e}}}}\left[{\widetilde{f}}_{{{{\rm{CM}}}}}\right]$ is generally lower at reduced frequencies, the intensified field concentration through microcapillaries generates a sufficiently high ∇|*E*|² to secure cell capture at lower applied voltages. Notably, the required capture voltage reached a minimum at 120 kHz, indicating an optimal physical trade-off between dielectric response (${{{\rm{R}}}}{{{\rm{e}}}}\left[{\widetilde{f}}_{{{{\rm{CM}}}}}\right]$) and spatial field confinement (∇|*E*|²). Thus, this optimized operational regime ensures robust, continuous single-cell capture at low voltages, effectively mitigating adverse effects such as Joule heating and undesired electrochemical reactions.

Operating precisely within this synergistic optimum (120 kHz), A549 cells experience strong pDEP forces and are securely guided to the capture sites, while WBCs are subjected to nDEP and are effectively repelled from the active regions. To experimentally validate this selective capability, we processed a mixed suspension of Calcein-AM-labeled A549 cells (green) and Hoechst 33342-labeled WBCs (blue). Figure 2f presents a representative trapping and separation event at a flow speed of 0.5 mm/s and an excitation of 71 *V*_rms_. As shown, A549 cells were selectively immobilized at the microcapillary orifices, while WBCs were continuously eluted by the hydrodynamic flow, achieving efficient single-cell isolation at 120 kHz. Interestingly, when the frequency was elevated to 150 kHz beyond the optimal operational window, both cell populations experienced pDEP and were attracted to the capillary openings, leading to non-selective capture. This observation confirms that our platform enables label-free selective capture by modulating the excitation frequency and voltage amplitude.

To better assess the DEP trapping performance, single-cell trapping efficiency was evaluated across various frequencies at a fixed excitation of 45 *V*_rms_ and a flow speed of 1 mm/s. Notably, our platform achieved an exceptional A549 single-cell trapping efficiency of >95% at 120 kHz (Fig. S6). This outperforms previously reported integrated DEP nanowell arrays (~92%)^40^ and an ITO-based PDMS configuration^41^. More importantly, while these conventional microchamber or planar electrode-based platforms inherently operate in a batch mode, often plagued by limited throughput and undesired cell clustering, our microcapillary device enables high-fidelity selective capture under continuous-flow conditions. This continuous-flow capability is exceptionally advantageous for isolating rare target cells, such as circulating tumor cells, from complex hematologic backgrounds. Interestingly, evaluating the voltage dependency at 120 kHz revealed a paradoxical decline in single-cell capture efficiency when the applied voltage exceeded 45 *V*_rms_ (Fig. S6b). This phenomenon is primarily driven by two factors: the onset of multi-cell co-trapping induced by excessively strong pDEP forces, and a potential compromise in cell viability caused by elevated electrical stress. The specific impacts of DEP parameters on cell viability and downstream transfection are systematically addressed in the subsequent section. Ultimately, by modulating the electric field, our platform seamlessly executes an automated “capture-processing-release” workflow. This dynamic control provides a versatile framework for downstream single-cell functional profiling, entirely circumventing the need for complex biochemical labeling or cumbersome off-chip sample transfer.

### Effects of DEP parameters on cellular viability and permeability

**Fig. 3:**
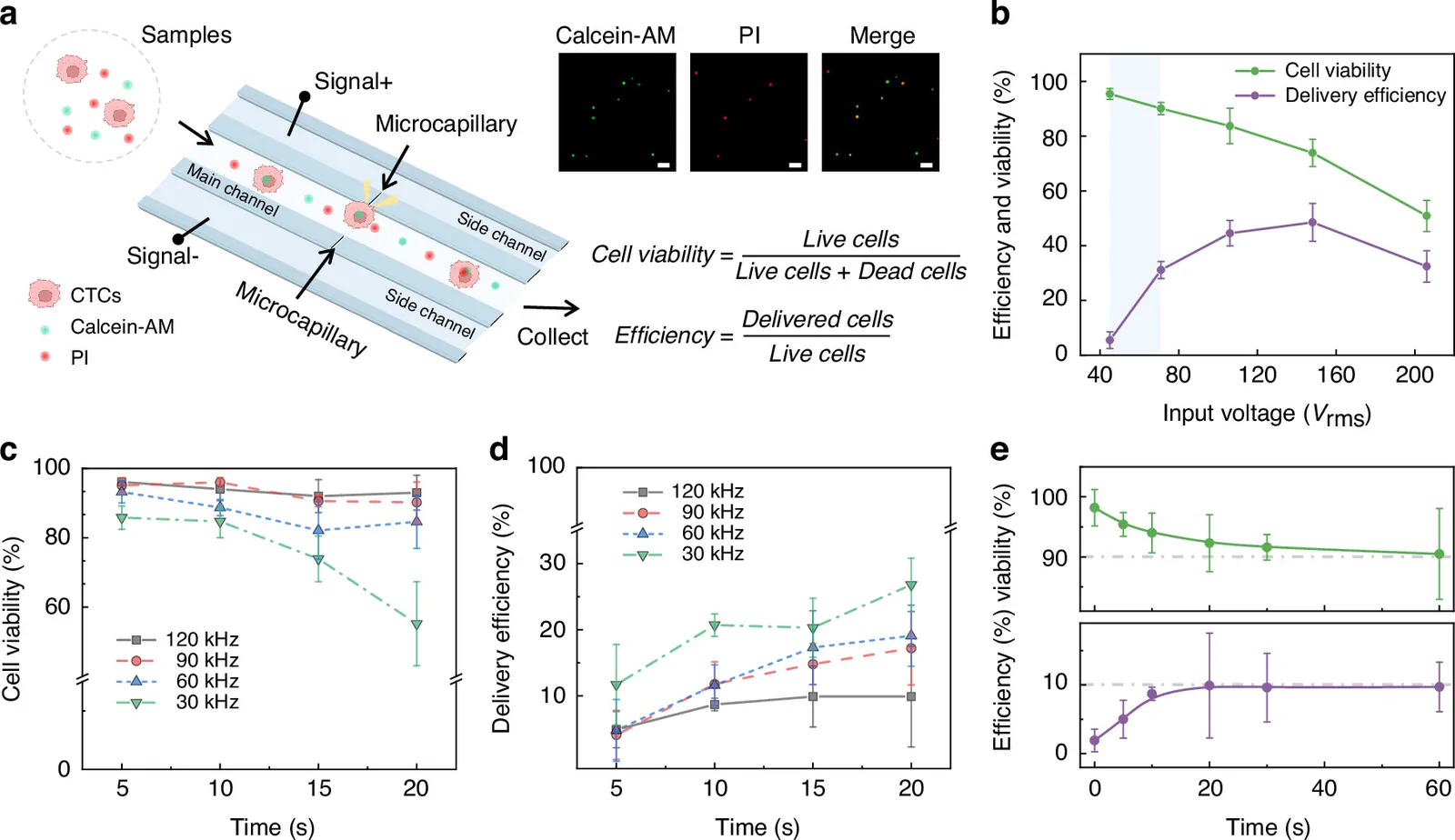
**Optimization of DEP parameters to preserve cell viability during single-cell trapping.** **a** Schematics illustrating the concurrent single-cell trapping and membrane permeabilization within the microcapillary device. Delivery efficiency and cell viability are quantitatively assessed using Calcein-AM and PI staining. Scale bars: 50 μm. **b** Delivery efficiency and cell viability as a function of the input voltage at a fixed frequency of 120 kHz. Characterization of A549 **c** cell viability and **d** delivery efficiency as a function of treatment duration at various excitation frequencies (30, 60, 90, and 120 kHz). **e** Long-term monitoring of delivery efficiency and cell viability measured at the optimized operational setting (45 *V*_rms_ and 120 kHz)

Although μC-DEP enables selective single-cell capture, the use of liquid electrodes inherently generates a high-intensity electrical field that risks irreversible membrane breakdown. Excessive applied voltage may cause severe cellular damage or mortality, thereby jeopardizing downstream applications such as gene delivery or single-cell profiling. Further, unintended membrane permeabilization and electroporation can occur concurrently during the DEP trapping phase. To systematically evaluate the impact of DEP parameters on cellular viability and membrane permeability, we performed a dual-fluorescence staining assay utilizing Calcein-AM and PI, as depicted in Fig. 3a. Calcein-AM, a membrane-permeable dye, is cleaved by intracellular esterases to emit green fluorescence, serving as a robust marker for viable cells. Conversely, PI is membrane-impermeable and exclusively intercalates into the nucleic acids of dead or permeabilized cells, emitting red fluorescence. This dual-staining strategy enables the precise discrimination among intact viable cells, dead cells, and transiently permeabilized cells.

We first investigated the impact of applied voltage on cell viability and membrane permeabilization by processing A549 cells pre-mixed with 10 μM Calcein-AM/PI. A 120 kHz sinusoidal signal was applied across the side channels for 5 s (Fig. 3b). The results revealed that within low voltage regimes (<71 *V*_rms_), the cells maintained excellent viability (>90%) but exhibited minimal uptake of exogenous dyes. As anticipated, the proportion of permeabilized cells correlated positively with the applied voltage. However, exceeding a threshold of 150 *V*_rms_ precipitated a sharp decline in cell viability due to irreversible membrane rupture. This massive cell death offset any gains in membrane permeability, severely diminishing the absolute number of successfully delivered cells and defining a strict upper limit for purely electro-mediated permeabilization. Notably, operating at the previously optimized capture voltage (45 *V*_rms_) preserved high viability while inducing negligible unintended permeabilization, thereby confirming it as a safe and efficient parameter for continuous cell sequestration. Building upon this, we interrogated the coupled effects of signal frequency and exposure duration (Fig. 3c, d). At a constant treatment time, decreasing the frequency markedly enhanced cargo uptake but triggered a concomitant plunge in cell viability. This trend robustly corroborates our numerical simulations, which confirm that lower frequencies concentrate the electric field gradient at the microcapillary orifices through conductive coupling. While this intensified and localized field drives membrane permeabilization, it simultaneously exacerbates the risk of irreversible membrane rupture, compromising cellular integrity. As illustrated in Fig. 3c, at a frequency of 30 kHz, prolonging the exposure duration caused severe cytotoxicity. In contrast, at 120 kHz, the cellular integrity remained robustly intact even under sustained electrical exposure. Continuous monitoring over 60 s at 120 kHz and 45 *V*_rms_ verified its exceptional biocompatibility, sustaining high viability while restricting baseline electroporation to <10% (Fig. 3e). These findings highlight frequency as the critical fulcrum balancing membrane permeabilization against cell survival. Although low-frequency excitation intensifies spatial field confinement to promote molecular uptake, this advantage is negated by severe cytotoxicity. Therefore, an excitation of 120 kHz at 45 *V*_rms_ establishes an optimal parametric window, ensuring stable cell trapping without functional impairment. Nevertheless, the inherently low intrinsic electroporation efficiency (<10%) under these benign conditions necessitates an auxiliary physical stimulus for robust intracellular delivery. In the following section, we elucidate how integrating localized acoustic waves elegantly resolves this bottleneck, triggering efficient sonoporation while fully preserving the biocompatibility of the DEP trapping field.

### Acoustical sonoporation for high-efficiency intracellular delivery

**Fig. 4:**
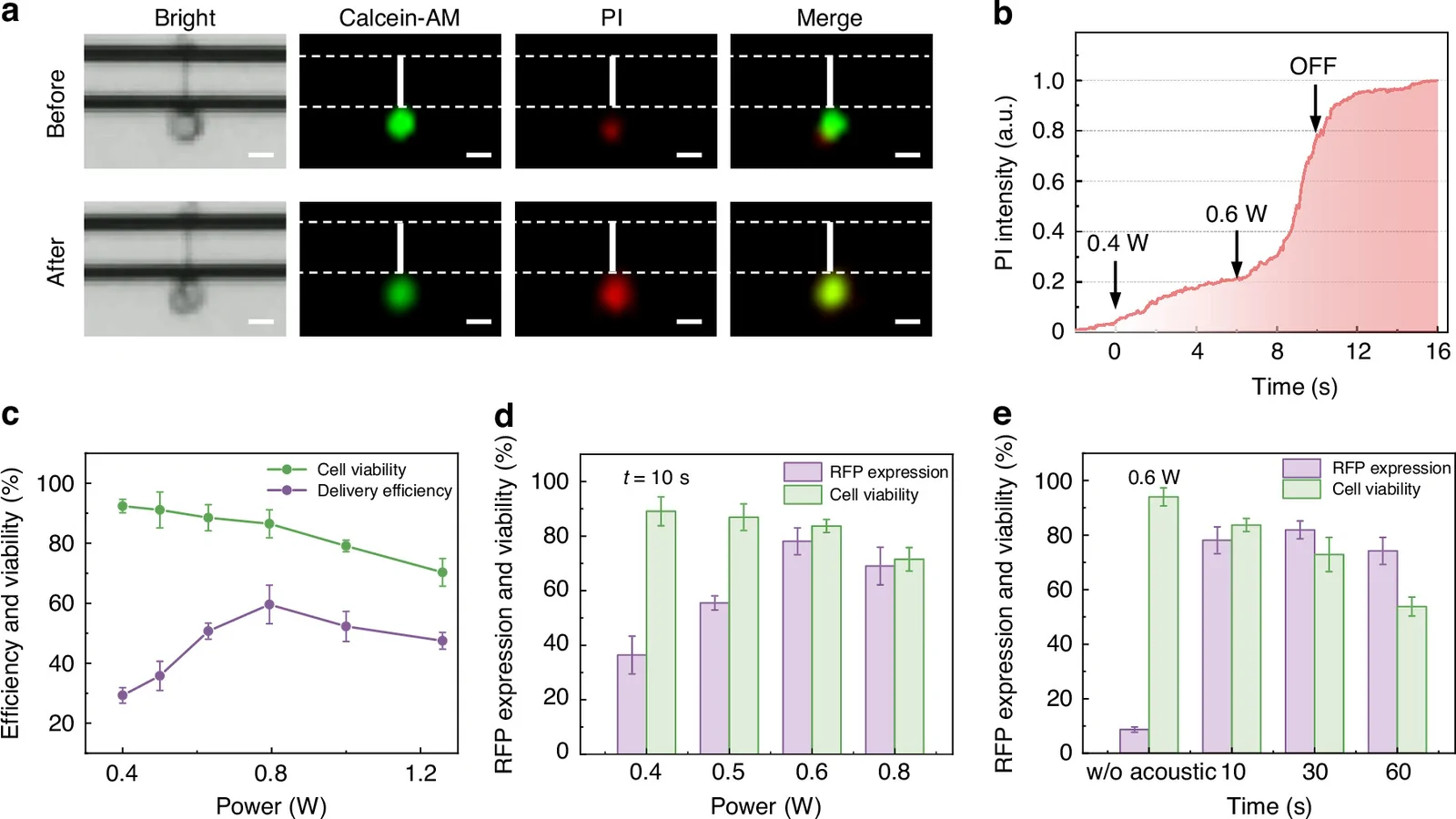
**On-site sonoporation enabled by μC-ASSIST platform for diverse biomolecular delivery.** **a** Fluorescence images of a single captured A549 cell before and after sonoporation. Green: Calcein-AM (viability); Red: PI (membrane permeabilization). Scale bars: 10 μm. **b** Quantitative analysis of the average intracellular PI intensity during SAW exposure at 0.4 W and 0.6 W. **c** Delivery efficiency (PI delivery) and cell viability of A549 cells as a function of the input acoustic power at a fixed 5 s SAW exposure. **d** RFP expression (mRFP-C3 delivery) and cell viability of A549 cells as a function of input acoustic power, at a fixed 10 s SAW exposure. **e** RFP expression and cell viability as a function of SAW exposure duration at a fixed 0.6 W input power

To enhance intracellular delivery without compromising cellular viability, we integrated SAW actuation on the chip to sonoporate the cells pre-captured within the microcapillaries. This acoustoelectric approach overcomes the limitations of permeabilization driven by electric fields alone. Cell viability and membrane integrity were monitored in real time using a Calcein-AM/PI dual-staining assay. Initially, a single A549 cell was selectively immobilized at the microcapillary orifice via DEP (120 kHz, 45 *V*_rms_). The emission of intense green fluorescence, resulting from intracellular Calcein-AM hydrolysis, confirmed that the cell remained metabolically active post-capture (Fig. 4a). After subsequent activation of SAW, the FIDT (50 MHz) triggered a rapid influx of PI, as evidenced by the progressive escalation of cytosolic red fluorescence. This temporal accumulation of PI directly visualizes the continuous molecular diffusion through the acoustically induced nanopores. Notably, the stable retention of green fluorescence alongside an intact cell morphology throughout the acoustic treatment indicated that the sonoporation process was transient and non-lethal, preserving the overall structural integrity of the cell. In contrast to conventional low-frequency ultrasound, the SAW excitation employed in the μC-ASSIST platform operates in the megahertz range (>10 MHz)^30^, which effectively suppresses violent inertial cavitation and minimizes collateral cellular damage. Instead, it induces reversible membrane deformation driven by the mechanical strain resulting from acoustic forces^42,43^. The magnitude of this deformation is governed by the acoustic pressure amplitude and excitation frequency; it intensifies with higher acoustic power but diminishes at elevated frequencies (see Section S2). This inherent frequency-dependent behavior explains the negligible cavitation-induced damage observed in our system, facilitating precise control over membrane permeability. As theoretically framed in Section S2 using the bilayer sonophore model, the transition from lethal rupture to reversible poration is governed by a frequency-dependent scaling law. The model posits that the maximal areal strain, which dictates irreversible membrane rupture, is inversely proportional to the excitation frequency. By operating in the MHz regime, our μC-ASSIST platform fundamentally suppresses this strain, bypassing the stochastic and destructive nature of cavitation. Furthermore, membrane permeabilization at these high frequencies is driven by direct coupling with the “soft modes” of the lipid bilayer. According to the Reusch model (see Section S2), SAWs induce collective structural oscillations in membrane thickness, facilitating the formation of localized, transient nanopores. Ultimately, this synergistic interplay between attenuated global strain and intensified molecular-level oscillation enables the precise modulation of membrane permeability while ensuring high cell viability.

To further elucidate the poration dynamics, we analyzed the time-lapse fluorescence intensity profiles. As depicted in Fig. 4b, the intracellular PI intensity exhibited a steady linear increase at the onset of SAW at 0.4 W (0–6 s), indicating a constant rate of molecular influx. Upon elevating the SAW power to 0.6 W (6–10 s), the fluorescence trajectory transitioned to a significantly steeper linear slope, reflecting an accelerated permeabilization rate driven by increased pore density or size. Immediately following the cessation of the SAW at 10 s, the intensity reached a stable plateau. This quick stabilization suggests that the membrane pores resealed rapidly once the acoustic driving force was removed, effectively sequestering the delivered molecular payload. This power-dependent modulation underscores the platform’s ability to precisely tune the delivery kinetics in real time. To establish the optimal operating regime, we statistically evaluated the performance across a broader cell population (*n* > 100) under varying acoustic intensities for a 5-s duration. As illustrated in Fig. 4c, the intracellular delivery efficiency correlated directly with acoustic power, increasing from 0.4 to 0.8 W while maintaining high cell viability (>85%). However, as the power exceeded this threshold (0.8 W), a marked decline in cell viability was observed, which consequently led to a reduction in the delivery efficiency. This performance peak suggests that beyond 0.8 W, the SAW-induced mechanical strain surpasses the elastic recovery limit of the lipid bilayer, resulting in irreversible membrane damage rather than transient poration. Furthermore, investigations into the exposure duration revealed that while prolonged acoustic treatments augmented molecular uptake, they concurrently led to a substantial decline in cell viability at 0.8 W (Fig. S7). This trend is likely attributed to the cumulative mechanical stress generated by sustained acoustic energy deposition. In contrast, operating at a moderate power of 0.6 W for 10 s yielded a high delivery efficiency (86.1 ± 3.05%) while preserving excellent cellular viability (85.5 ± 5.76%). Although further prolonging the exposure time to 15 s marginally enhanced the efficiency, it incurred a pronounced penalty to cell viability. Consequently, the combination of 0.6 W and 10 s was established as the optimal condition for small-molecule delivery, striking an ideal equilibrium between maximized delivery efficiency and robust cellular survival.

Following the successful validation of small-molecule delivery, we advanced the platform’s utility to the intracellular transport of macromolecular cargo, specifically targeting plasmid DNA for functional nuclear expression. The mRFP-C3 plasmid was employed as a model macromolecule. Unlike the simple passive diffusion of small dyes, the successful delivery and subsequent expression of large plasmids require the formation of sufficiently large, transient pores alongside the maintenance of post-poration cellular machinery. To demonstrate the scalability and augment throughput of the μC-ASSIST platform, we implemented an expanded architecture featuring an array of 15 microcapillary pairs (Fig. S8). This parallelized configuration allows for the simultaneous capture and transfection of multiple single cells within a uniform acoustoelectric field. As depicted in Fig. S8, the device maintained high uniformity in both capture efficiency and molecular uptake across the entire array, confirming that our synergistic DEP-SAW mechanism scales robustly without sacrificing the fidelity of single-cell manipulation. To evaluate the platform’s capacity for genetic modification, A549 cells were suspended in a mannitol solution supplemented with 20 μg/mL mRFP-C3 plasmid and introduced into the μC-ASSIST device. Following the synergistic DEP-SAW treatment, the retrieved cells underwent a 4-h recovery incubation to facilitate membrane resealing and metabolic restoration. After a subsequent washing step to remove extracellular plasmids, the cells were cultured for 48 h to allow for comprehensive protein expression. The emergence of robust red fluorescence (Fig. S9) definitively validates the successful internalization, nuclear translocation, and functional expression of the mRFP-C3 plasmid. This underscores the platform’s efficacy to deliver large macromolecular cargo while maintaining the long-term cellular homeostasis necessary for complex biological processes. We subsequently fine-tuned the device parameters for plasmid transfection. At a constant SAW exposure of 10 s (Fig. 4d), the RFP expression efficiency increased consistently as power was raised from 0.4 to 0.6 W, but subsequently declined at 0.8 W due to compromised cellular viability. This biphasic trend mirrors the permeabilization kinetics previously observed with PI, indicating a universal parametric response across different molecular scales. Under the optimized parameters (0.6 W, 10 s), the RFP expression efficiency peaked at 78. 1 ±  4.9%, concomitantly maintaining a high cell viability of 83.7  ± 2.4% (Fig. 4e). Notably, extending the exposure beyond 10 s precipitated a severe drop in viability. Collectively, these results establish 0.6 W and 10 s exposure as the optimal conditions for plasmid transfection, achieving high delivery efficiency while rigorously preserving cellular integrity. Compared with existing acoustic-mediated platforms (Table S3), the μC-ASSIST system delivers superior transfection performance utilizing significantly lower power inputs and shorter exposure durations, all while uniquely enabling label-free, highly selective single-cell manipulability.

### Targeted gene silencing and functional inhibition of tumor cell motility

**Fig. 5:**
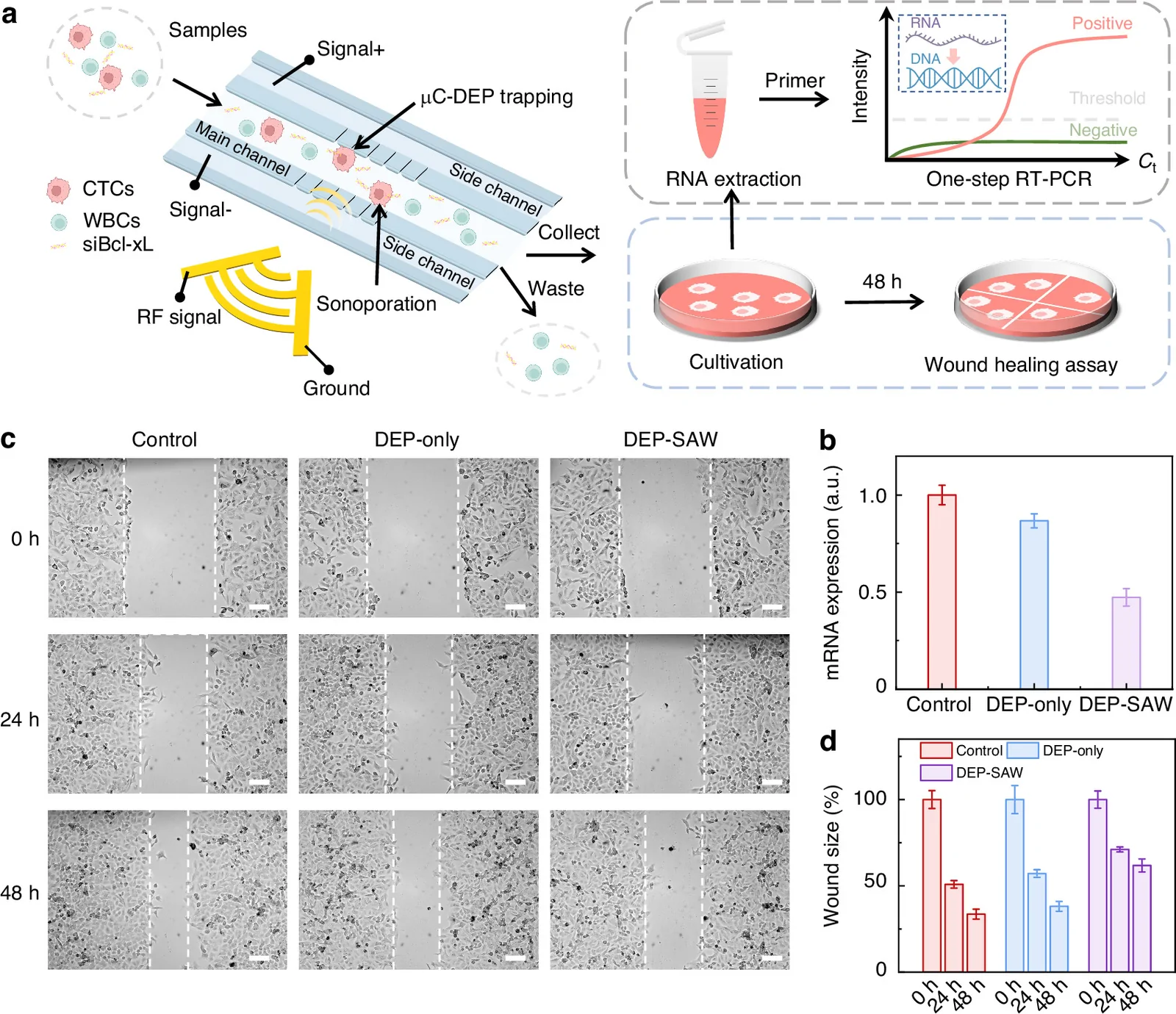
**Functional siRNA delivery and proof-of-concept inhibition of tumor cell migration using the μC‑ASSIST platform.** **a** Schematic workflow for integrated separation and transfection in a complex matrix. A549 cells are isolated and transfected with siRNA via on-site sonoporation, followed by downstream RT-qPCR and wound healing assays. **b** Relative Bcl-xL mRNA expression levels quantified by RT-qPCR 48 h post siBcl-xL delivery. **c** Representative images from the wound healing assay showing the inhibition of A549 cell migration across the three treatment groups (Control, DEP-only, and DEP-SAW) at 0, 24, and 48 h. Scale bars: 100 μm. **d** Quantification of the wound size over the 48-h period

Leveraging the optimal operating parameters, we validated the platform’s capacity for siRNA delivery by introducing Cy3‑labeled GAPDH siRNA into A549 cells. Fluorescence microscopy revealed a robust and uniform intracellular accumulation of Cy3 signals, achieving a high delivery efficiency of 83.2 ± 6.3% (Fig. S10). This efficient uptake of fluorescent oligonucleotides confirms that the μC-ASSIST platform provides a sufficiently expansive and stable poration window to accommodate short-chain nucleic acids. To further evaluate the translational potential of the μC-ASSIST platform, we performed functional gene-silencing studies targeting the anti-apoptotic gene Bcl-xL (siBcl-xL). Bcl-xL inhibition is known to destabilize the mitochondrial membrane and elevate intracellular reactive oxygen species, a pathway critical for suppressing tumor invasion^33,34^. To evaluate the selective isolation and downstream transfection capabilities within a heterogeneous background, A549 cells were spiked into WBCs at a 1:100 ratio, serving as an in vitro model of a pre-enriched cell suspension. Three parallel treatment groups were analyzed: (1) Control (infusion only); (2) DEP-only (capture without SAW); and (3) DEP-SAW (integrated capture and sonoporation) (Fig. 5a). Following cell collection and incubation, Bcl-xL mRNA expression levels were quantified using real-time quantitative reverse transcription polymerase chain reaction (qRT-PCR), as shown in Fig. 5b. Compared to the control and DEP-only groups, the DEP-SAW group exhibited a significant downregulation in mRNA expression (52.7% knockdown), validating successful siRNA delivery and effective gene silencing. The subsequent phenotypic impact of Bcl‑xL silencing on cell motility was evaluated via a wound healing assay (Fig. 5c). While all groups presented comparable wound widths at 0 h, distinct migratory behaviors diverged over a 48-h observation period. At 24 h, partial wound closure was observed across all cohorts, likely attributable to a combination of basal cell proliferation and stochastic migration. However, the inhibitory effect became pronounced at 48 h. As illustrated in Fig. 5d, the DEP-SAW group displayed markedly suppressed migration, retaining a significantly larger unhealed wound area (61.84 ± 3.79%) compared to the control (33.64 ± 2.88%) and DEP‑only (38.15 ± 2.84%) groups. This corresponds to a ~50% reduction in the migration rate (Fig. S11). These findings confirm that the μC-ASSIST platform enables not only high-efficiency siRNA delivery but also precise, gene-specific functional modulation, offering great opportunities for targeted cancer gene therapy and therapeutic screening.

## Conclusion

In this study, we introduce μC-ASSIST, a synergistic microfluidic platform that seamlessly integrates μC-DEP with high-frequency SAWs to achieve the selective capture and high-efficiency transfection of single target cells. The hallmark of this platform is its spatial selectivity. By exploiting localized non-uniform electric fields at the microcapillary orifices, we realized the high-fidelity isolation of specific tumor cells from heterogeneous matrices. This DEP-driven pre-selection step ensures that subsequent therapeutic cargoes are delivered exclusively to the cells of interest, thereby eliminating the risk of off-target transfection in mixed populations, which is an imperative prerequisite for clinical liquid biopsy applications. The subsequent delivery phase utilizes high-frequency SAWs to bridge the gap between safety and efficiency. Distinct from conventional electroporation or low-frequency sonoporation, our platform uniquely activates the soft modes of the lipid bilayer while suppressing lethal inertial cavitation. This allows for the formation of transient, reversible pores, achieving an optimal balance of 78.1% transfection efficiency and 83.7% cell viability. The dynamic nature of these pores, demonstrated by real-time fluorescence kinetics, underscores the system’s high temporal precision. The platform’s functional utility was validated through the effective delivery of mRFP-C3 plasmids and the functional silencing of the Bcl-xL gene, which resulted in a 50% reduction in tumor cell motility. The capacity to execute complex, targeted genetic manipulations on rare cells within a scalable, parallelized microcapillary array establishes the μC-ASSIST platform as a versatile tool for personalized oncology and functional genomics. Moving forward, integrating this platform with automated, real-time feedback mechanisms will firmly solidify its role as a standardized instrument for precision single-cell engineering.

## Materials and methods

### Device design and fabrication

The μC-ASSIST chip for single-cell selective trapping and intracellular delivery was comprised of a PDMS slab with embedded microcapillaries, a PDMS coupling layer, and a LiNbO_3_ substrate patterned with an FIDT. The master mold for the PDMS slab was fabricated through a double-layer photolithography process (Fig. S4). First, AZ-5214 photoresist was spin-coated on a silicon wafer and patterned via a reversal baking protocol, followed by inductively coupled plasma (ICP) dry etching to create microscale ridge structures (2 μm width × 2 μm height × 20 μm length). Subsequently, a layer of 50 μm-thick SU-8 3050 photoresist was patterned on the etched substrate to form microchannels, including a 50 μm-wide main channel and two 30 μm-wide side channels. Before PDMS replication, the silicon mold underwent trichloro(1H,1H,2H,2H-perfluorooctyl) silane (Sigma-Aldrich, Missouri, USA) vapor treatment for 30 min to facilitate demolding. The PDMS base and curing agents (Sylgard 184, Dow Corning, MI) were mixed in a 10:1 (w/w) ratio, degassed in a vacuum chamber, poured onto the mold, and cured at 80 °C for 2 h. After curing, the slab containing microfluidic channels was carefully peeled off, and holes were punched for the inlet and outlet. The remaining PDMS mixture was spin-coated onto a clean silicon wafer to form a thin coupling layer (20–30 μm thick), which was then cured and peeled off for later use. The microchannel layer and the coupling layer were bonded via oxygen plasma treatment (60 W, 30 s; PT‑15S, Potentlube, Shenzhen, China). The FIDT was fabricated by patterning the structures of 150 nm-thick Al on a LiNbO_3_ wafer via standard photolithography and a lift-off process. The FIDT comprises 25 pairs of fingers with a uniform width and spacing of 20 μm (*λ*/4), yielding a resonance frequency of 50 MHz. The focusing angle and focal length of the FIDT are 60° and 500 μm, respectively. For final assembly, the microfluidic channel was aligned and bonded to the LiNbO₃ substrate. In this assembled structure, the PDMS coupling layer acts as a robust dielectric barrier, isolating the liquid electrode from the piezoelectric substrate. This structural dielectric isolation, together with the large separation between the 120 kHz DEP excitation and the 50 MHz FIDT resonance, is expected to minimize unintended electrical and electromechanical coupling between the two functional modules.

### Numerical computation and simulation

The microcapillary structure was designed using AutoCAD software and subsequently imported into COMSOL Multiphysics software for finite element analysis. The electric potential distribution was solved through the Laplace equation $\nabla \cdot \left({\sigma }^{* }\nabla \phi =0\right)$, with the boundary condition (E=0) applied at electrode interfaces. Material properties were assigned as follows: liquid electrodes (σ = 200 mS/cm), sample medium (σ = 100 μS/cm), and PDMS (σ= 0.8 × 10^−8^ μS/cm). Relative permittivity values were set to 80 for aqueous solution and 2.65 for PDMS. Field characteristics, including electric field intensity ($E=-\nabla \phi$) and field gradient ($\nabla E=\nabla (-\nabla \phi )$), were derived computationally to quantify DEP response dynamics.

The direction of DEP forces was determined by the real component of the ${{{\rm{R}}}}{{{\rm{e}}}}[{\widetilde{f}}_{{{{\rm{CM}}}}}]$. Frequency-dependent CM factor analysis for A549 cells and WBCs was performed over 10^3^–10^8 ^Hz using a medium relative permittivity of 78 and conductivities of 10, 100, and 1000 μS/cm, respectively. The cell parameters are listed in Table S2.

### Cell culture and sample preparation

A549 cells (Beyotime Biotechnology, Shanghai, China) were cultured in high-glucose Dulbecco’s Modified Eagle Medium (DMEM; Thermo Fisher Scientific, USA) supplemented with 10% fetal bovine serum (FBS; Thermo Fisher Scientific, USA) and 1% penicillin-streptomycin (Gibco, USA). The cells were maintained at 37 °C in a humidified incubator with 5% CO_2_. For harvesting, adherent cells were washed with phosphate-buffered saline (PBS; Biosharp, China) and detached using 0.25% trypsin-EDTA solution (Sigma-Aldrich, USA). Following centrifugation, A549 cells were resuspended in 1 × PBS solution (~1 × 10^6^ cells/mL) for subsequent experiments. Blood specimens were drawn from healthy donors after obtaining informed consent with ethical approval from Zhejiang University (ZJU202160) and collected into EDTA-coated vacutainer tubes (BD Biosciences, USA). To prepare the WBC samples, whole blood was mixed with a three-fold volume of erythrocyte lysis buffer (Beyotime, China) in a centrifuge tube and incubated at ambient temperature for 5–10 min. After centrifugation and removal of the supernatant, WBCs were collected and resuspended using 1 × PBS solution (~1 × 10^6^ cells/mL) for subsequent experiments.

To prepare the working fluids for the microfluidic device, a high-conductivity buffer as the liquid electrode buffer was prepared by supplementing a concentrated 10 × PBS solution with NaCl to achieve a conductivity of 200 mS/cm. To minimize Joule heating and maintain cell viability during DEP and SAW operations, a specific cell buffer (low conductivity) was prepared by dissolving 300 mM D-mannitol in deionized water and adjusted to a final conductivity of 100 μS/cm by adding 1 × PBS.

### Microfluidic single-cell capture and on-chip delivery

For single-cell selective capture experiments, A549 cells and WBCs were pre-stained with Calcein-AM (Yeasen, China) and Hoechst 33342 (Beyotime, China), respectively, according to the manufacturer’s protocols. The cells were washed twice and resuspended in the low-conductivity cell buffer. To assess the device capture selectivity in a heterogeneous cell mixture, A549 cells were spiked into the WBC suspension at a ratio of 1:100.

For on‑chip delivery and sonoporation studies, different biomolecules were introduced into A549 cell suspensions prior to chip injection. Propidium iodide (PI; Yeasen, China) was added at a final concentration of 10 μg/mL to serve as an indicator of membrane permeabilization. For plasmid delivery, mRFP‑C3 was diluted in the cell buffer to a final concentration of 20 μg/mL and pre-mixed with the cell suspension. For siRNA delivery, either Cy3‑labeled GAPDH siRNA (Thermo Fisher Scientific, USA) or siBcl‑xL (20 nM; Puhe Bio-Technology, China) was mixed with the cell suspension before processing.

The cell mixture was introduced into the main channel of the μC-ASSIST chip at a flow speed of 1 mm/s, while the liquid electrode buffer was injected into the side channels. Optimized DEP parameters (45 *V*_rms_, 120 kHz) were applied to trap single cells, followed by SAW excitation (0.6 W, 10 s) to induce sonoporation.

### Characterization of intracellular delivery, transfection efficiency, and cell viability

Acute membrane permeabilization after various treatments was immediately assessed using fluorescence microscopy (Nikon FN1, Japan). Calcein-AM (green fluorescence) was utilized to identify viable cells, while PI (red fluorescence) uptake served as the indicator of successful membrane permeabilization. Using this dual-staining scheme, the delivery efficiency was calculated as the ratio of double-positive cells (Green+/Red+) to the total number of viable cells (Green+). Concurrently, cell viability was determined by comparing the number of viable cells (Green+) to the total cell count (*N*_live_ + *N*_dead_), where *N*_dead_ refers to PI-only positive cells.

For long-term viability and reporter gene expression studies involving plasmid (mRFP-C3) and siRNA transfection, the collected cells were incubated in 96-well plates for 48 h post-treatment. RFP expression, indicating successful plasmid delivery and expression, was quantified by counting cells exhibiting red fluorescence signals relative to the total number of cells observed under the fluorescence microscope. Separately, long-term cell viability was assessed using the Cell Counting Kit-8 (CCK-8; Beyotime, China). The assay involved measuring absorbance (OD) at 450 nm using a microplate reader (Spark, Tecan, Switzerland). Relative cell viability was calculated by normalizing the background-subtracted absorbance of the experimental group to that of the untreated control well.

### Quantitative real-time PCR and wound healing assay

To evaluate gene silencing efficiency, total RNA was extracted from collected cells 48 h post-transfection using RNAiso Plus (Takara, Japan) following the manufacturer’s strict RNase-free protocols. RNA concentration and purity (OD_260_/OD_280_ ratio ~1.8–2.0) were verified using a spectrophotometer (RF-6000, Shimadzu, China). Reverse transcription and PCR amplification were performed in a single step using the One Step PrimeScript™ RT-PCR Kit (Takara, Japan) on a Q1000 Real-Time PCR System (LongGene, China). Primers and probes for Bcl-xL (target gene) and GAPDH (internal reference) were synthesized by Puhe Bio-Technology (Shanghai, China). The primer sequences are listed in Table S4. Relative mRNA expression levels were calculated using the comparative Ct method (${2}^{-\triangle \triangle {C}_{t}}$).

The migratory capacity of A549 cells following siBcl-xL treatment was assessed using a wound healing assay. Cells collected from the μC-ASSIST chip were seeded into 24-well plates and cultured until forming a confluent monolayer. The monolayer was mechanically scratched using a sterile 200 μL pipette tip to create a cell-free gap. The wells were washed once with PBS to remove cell debris and replenished with fresh serum-free medium. Wound closure was monitored by capturing images at 0, 24, and 48 h using an inverted microscope. The unhealed wound area was quantified using ImageJ software (NIH, USA). The relative wound size was calculated as *A*_t_/*A*_0_ × 100%, where *A*_0_ is the initial wound area at *t* = 0 h, and *A*_t_ is the wound area at the specified time point.

### Data analysis

All quantitative data are presented as mean ± standard deviation from at least three independent experiments (*n* ≥ 3). The average fluorescence intensity of the cell was measured and processed by ImageJ. The relative fluorescence intensity was calculated by normalizing the average cellular fluorescence to the background fluorescence.

## Supplementary information

Supporting Information

## References

[1] Lawrence, R., Watters, M., Davies, C. R., Pantel, K. & Lu, Y.-J. Circulating tumour cells for early detection of clinically relevant cancer. *Nat. Rev. Clin. Oncol.* **20**, 487–500 (2023). 10.1038/s41571-023-00781-y PMC10237083 37268719

[2] Bergers, G. & Fendt, S.-M. The metabolism of cancer cells during metastasis. *Nat. Rev. Cancer* **21**, 162–180 (2021). 10.1038/s41568-020-00320-2 PMC8733955 33462499

[3] Stewart, C. A. et al. Single-cell analyses reveal increased intratumoral heterogeneity after the onset of therapy resistance in small-cell lung cancer. *Nat. Cancer* **1**, 423–436 (2020). 10.1038/s43018-019-0020-z PMC7842382 33521652

[4] Keller, L. & Pantel, K. Unravelling tumour heterogeneity by single-cell profiling of circulating tumour cells. *Nat. Rev. Cancer* **19**, 553–567 (2019). 10.1038/s41568-019-0180-2 31455893

[5] Jackson, H. W. et al. The single-cell pathology landscape of breast cancer. *Nature* **578**, 615–620 (2020). 10.1038/s41586-019-1876-x 31959985

[6] Zhang, X. et al. Multiplex profiling of biomarker and drug uptake in single cells using microfluidic flow cytometry and mass spectrometry. *ACS Nano* **18**, 6612–6622 (2024). 10.1021/acsnano.3c12803 PMC10906074 38359901

[7] Wang, C. et al. Microfluidic biochips for single-cell isolation and single-cell analysis of multiomics and exosomes. *Adv. Sci.* **11**, 2401263 (2024). 10.1002/advs.202401263 PMC11267386 38767182

[8] Lan, Y., Zhou, Y., Wu, M., Jia, C. & Zhao, J. Microfluidic based single cell or droplet manipulation: methods and applications. *Talanta* **265**, 124776 (2023). 10.1016/j.talanta.2023.124776 37348357

[9] Stöcklin, L. R., Dietsche, C. L. & Dittrich, P. S. A high-density microchamber array for the analysis of extracellular vesicles derived from single cells under drug treatment. *Anal. Chem.* **98**, 1460–1469 (2026). 10.1021/acs.analchem.5c05621 PMC12824987 41489814

[10] Shembekar, N., Hu, H., Eustace, D. & Merten, C. A. Single-cell droplet microfluidic screening for antibodies specifically binding to target cells. *Cell Rep.* **22**, 2206–2215 (2018). 10.1016/j.celrep.2018.01.071 PMC5842027 29466744

[11] Wu, B. et al. High-throughput microfluidic production of droplets and hydrogel microspheres through monolithically integrated microchannel plates. *Anal. Chem.* **95**, 13586–13595 (2023). 10.1021/acs.analchem.3c02250 37624148

[12] Li, G. et al. Massive and controllable production of droplets on chips induced by surface acoustic waves for multiplex digital PCR detection of bacteria. *Sens. Actuators B Chem.* **446**, 138763 (2026).

[13] Zheng, T., Zhang, Z. & Zhu, R. Flexible trapping and manipulation of single cells on a chip by modulating phases and amplitudes of electrical signals applied onto microelectrodes. *Anal. Chem.* **91**, 4479–4487 (2019). 10.1021/acs.analchem.8b05228 30860356

[14] Chen, H. et al. Quantification of capture efficiency, purity, and single-cell isolation in the recovery of circulating melanoma cells from peripheral blood by dielectrophoresis. *Lab Chip* **23**, 2586–2600 (2023). 10.1039/d2lc01113a PMC10228177 37185977

[15] Guo, F. et al. Three-dimensional manipulation of single cells using surface acoustic waves. *Proc. Natl. Acad. Sci. USA.* **113**, 1522–1527 (2016). 10.1073/pnas.1524813113 PMC4760790 26811444

[16] Ahmed, D. et al. Rotational manipulation of single cells and organisms using acoustic waves. *Nat. Commun.* **7**, 11085 (2016). 10.1038/ncomms11085 PMC4814581 27004764

[17] Yang, S. et al. Acoustic tweezers for advancing precision biology and medicine. *Nat. Rev. Method Primers* **5**, 49 (2025).

[18] Labib, M. et al. Magnetic ranking cytometry: profiling rare cells at the single-cell level. *Acc. Chem. Res.* **53**, 1445–1457 (2020). 10.1021/acs.accounts.0c00179 32662263

[19] Guo, X. et al. Design of an automated cell batch microinjection system based on magnetic tweezers for zebrafish embryos. *Microsyst. Nanoeng.* **12**, 113 (2026). 10.1038/s41378-026-01230-3 PMC13036081 41912488

[20] Duckert, B., Vinkx, S., Braeken, D. & Fauvart, M. Single-cell transfection technologies for cell therapies and gene editing. *J. Control. Release* **330**, 963–975 (2021). 10.1016/j.jconrel.2020.10.068 33160005

[21] Hur, J. & Chung, A. J. Microfluidic and nanofluidic intracellular delivery. *Adv. Sci.* **8**, 2004595 (2021). 10.1002/advs.202004595 PMC8336510 34096197

[22] Stewart, M. P. et al. In vitro and ex vivo strategies for intracellular delivery. *Nature* **538**, 183–192 (2016). 10.1038/nature19764 27734871

[23] Cao, Y. et al. Nontoxic nanopore electroporation for effective intracellular delivery of biological macromolecules. *Proc. Natl. Acad. Sci. USA.* **116**, 7899–7904 (2019). 10.1073/pnas.1818553116 PMC6475394 30923112

[24] Li, K. et al. Bipolar electrode-based precise manipulation and selective electroporation of cells. *Anal. Chem.* **97**, 3908–3919 (2025). 10.1021/acs.analchem.4c05041 39950441

[25] Sun, Y. et al. Recent advances in micro/nanoneedle arrays mediated intracellular delivery of biomacromolecules in vitro and in vivo. *Adv. Funct. Mater.* **35**, 2422234 (2025).

[26] Li, X. et al. High-throughput and efficient intracellular delivery method via a vibration-assisted nanoneedle/microfluidic composite system. *ACS Nano.* **17**, 2101–2113 (2022). 10.1021/acsnano.2c07852 36479877

[27] Sevenler, D. & Toner, M. High throughput intracellular delivery by viscoelastic mechanoporation. *Nat. Commun.* **15**, 115 (2024). 10.1038/s41467-023-44447-w PMC10762167 38167490

[28] Li, Z. et al. Acoustofluidics-Based Intracellular Nanoparticle Delivery. *Engineering* **47**, 130–138 (2025). 10.1016/j.eng.2024.11.030 PMC12054690 40330125

[29] Liu, X. et al. Acoustothermal transfection for cell therapy. *Sci. Adv.* **10**, eadk1855 (2024). 10.1126/sciadv.adk1855 PMC11023511 38630814

[30] Ramesan, S., Rezk, A. R., Dekiwadia, C., Cortez-Jugo, C. & Yeo, L. Y. Acoustically-mediated intracellular delivery. *Nanoscale* **10**, 13165–13178 (2018). 10.1039/c8nr02898b 29964280

[31] Luo, Y., Cao, X., Huang, P. & Yobas, L. Microcapillary-assisted dielectrophoresis for single-particle positioning. *Lab Chip* **12**, 4085–4092 (2012). 10.1039/c2lc40150a 22892643

[32] Wu, Y. et al. SonoPIN enables precise, noninvasive, and efficient intracellular delivery of PROTACs. *Proc. Natl. Acad. Sci. USA.* **123**, e2534439123 (2026). 10.1073/pnas.2534439123 PMC12993956 41824501

[33] Shen, Q. et al. Sensitizing non-small cell lung cancer to BCL-xL-targeted apoptosis. *Cell Death Dis.* **9**, 986 (2018). 10.1038/s41419-018-1040-9 PMC6155218 30250075

[34] Zhang, M. et al. Programmed nanocarrier loaded with paclitaxel and dual-siRNA to reverse chemoresistance by synergistic therapy. *Int. J. Biol. Macromol.* **261**, 129726 (2024). 10.1016/j.ijbiomac.2024.129726 38290632

[35] Liang, C.-C., Park, A. Y. & Guan, J.-L. In vitro scratch assay: a convenient and inexpensive method for analysis of cell migration in vitro. *Nat. Protoc.* **2**, 329–333 (2007). 10.1038/nprot.2007.30 17406593

[36] Tian, Z., Wang, X. & Chen, J. On-chip dielectrophoretic single-cell manipulation. *Microsyst. Nanoeng.* **10**, 117 (2024). 10.1038/s41378-024-00750-0 PMC11347631 39187499

[37] Voldman, J. Electrical Forces for Microscale Cell Manipulation. *Annu. Rev. Biomed. Eng.* **8**, 425–454 (2006). 10.1146/annurev.bioeng.8.061505.095739 16834563

[38] Tada, S., Eguchi, M. & Okano, K. Insulator-based dielectrophoresis combined with the isomotive AC electric field and applied to single cell analysis. *Electrophoresis* **40**, 1494–1497 (2019). 10.1002/elps.201800380 30672595

[39] Islam, M. N., Jaiswal, B. & Gagnon, Z. R. High-throughput continuous free-flow dielectrophoretic trapping of micron-scale particles and cells in paper using localized nonuniform pore-scale-generated paper-based electric field gradients. *Anal. Chem.* **96**, 1084–1092 (2024). 10.1021/acs.analchem.3c03740 PMC10809225 38194698

[40] Bai, Z. et al. An integrated dielectrophoresis-trapping and nanowell transfer approach to enable double-sub-poisson single-cell RNA sequencing. *ACS Nano* **14**, 7412–7424 (2020). 10.1021/acsnano.0c02953 32437127

[41] Pendharkar, G., Lu, Y.-T., Chang, C.-M., Lu, M.-P. & Liu, C.-H. A high throughput biocompatible insulator based dielectrophoretic (iDEP) lab chip for patterning and fusion of biological cells. *Sens. Actuators B Chem.* **354**, 131109 (2022).

[42] Reusch, T. et al. Collective lipid bilayer dynamics excited by surface acoustic waves. *Phys. Rev. Lett.* **113**, 118102 (2014). 10.1103/PhysRevLett.113.118102 25260008

[43] Krasovitski, B., Frenkel, V., Shoham, S. & Kimmel, E. Intramembrane cavitation as a unifying mechanism for ultrasound-induced bioeffects. *Proc. Natl. Acad. Sci. USA.* **108**, 3258–3263 (2011). 10.1073/pnas.1015771108 PMC3044354 21300891

