# Supplementary material for: Microcapillary-assisted acoustoelectric platform for selective single-cell isolation and in situ transfection
Source: Microsyst Nanoeng. 2026 Oct 19;12:350. doi: 10.1038/s41378-026-01490-z (PMC13680384; doi:10.1038/s41378-026-01490-z)
Supplement: Supplementary file 1 [file 41378_2026_1490_MOESM1_ESM.docx]

**Supporting Information**

**Microcapillary-assisted acoustoelectric platform for selective single-cell isolation and *in situ* transfection**

Xuefeng Xu^1,2,†^, Ziqiao Zhou^2,†^, Guangyang Li^1,2^, Jie Liang^1,2^, Jianhui Wu^1,2^, Jiaqi Lu^1,2^, Fang Shen^6^, Shengjun Wu^4,5,*^, Zhi Ruan^4,5,*^, Shurong Dong^1,2^, Jikui Luo^1,2^ and Zhen Cao^1,2,3,5*^

^1^College of Information Science and Electronic Engineering, Zhejiang University, Hangzhou 310027, China

^2^International Research Center for Information Science and Electronic Engineering, Zhejiang University, Haining 314400, China

^3^Zhejiang Key Laboratory of Advanced Micro-Nano Transducers Technology, Zhejiang University, Shaoxing 312000, China

^4^Department of Clinical Laboratory, Sir Run Run Shaw Hospital, Zhejiang University School of Medicine, Hangzhou 310016, China

^5^Zhejiang Provincial Engineering Research Center of Innovative Instruments for Precise Pathogen Detection, Hangzhou 310016, China

^6^Department of Clinical Laboratory, Shaoxing Second Hospital, Shaoxing, China

*Address correspondence to [eezcao@zju.edu.cn](mailto:eezcao@zju.edu.cn); 3202107@zju.edu.cn; [r_z@zju.edu.cn](mailto:r_z@zju.edu.cn)

**The file includes:**

Section S1 and S2

Fig. S1 to S11

Table S1 to S4

**Section S1. Separation theory of the DEP force**

Dielectrophoresis (DEP) describes the translational motion of uncharged but polarizable particles due to the interaction between an applied non-uniform electric field and their induced dipole moments. For a spherical cell with radius *a* suspended in a medium, the time-averaged DEP force under a non-uniform electric field can be expressed as^1,2^

$$F_{\mathrm{DEP}}=2\pi\varepsilon_{m}a^{3}\mathrm{Re} \left[ \tilde{f}_{\mathrm{CM}} \right]\nabla\left| \vec{E} \right|^{2}$$

(1)

where $\varepsilon_{m}$ represents the absolute permittivity of the suspended medium, $\nabla\left| \vec{E} \right|^{2}$is the gradient of the square of the electric field. The direction of the DEP force was determined by the real component of the Clausius-Mossotti (CM) factor $\tilde{f}_{\mathrm{CM}}$, calculated as

$$\tilde{f}_{\mathrm{CM}}=\frac{\tilde{\varepsilon}_{P}-\tilde{\varepsilon}_{m}}{\tilde{\varepsilon}_{P}+2\tilde{\varepsilon}_{m}}$$

(2)

The complex permittivity $\tilde{\varepsilon}$ for either the particle $\tilde{\varepsilon}_{P}$ or the medium $\tilde{\varepsilon}_{m}$ is given by:

$$\tilde{\varepsilon}=\varepsilon-\frac{\sigmaⅈ}{w}$$

(3)

where $\varepsilon$ is the absolute permittivity, $\sigma$ the electrical conductivity, and $w$ the angular frequency of the applied electric field. When $\mathrm{Re} \left[ \tilde{f}_{\mathrm{CM}} \right]>0$, the cells experience positive DEP (pDEP) and migrate toward regions of high electric field gradients (*e.g.*, microcapillary orifices). Conversely, when $\mathrm{Re} \left[ \tilde{f}_{\mathrm{CM}} \right]<0$, cells experience negative DEP (nDEP) and are repelled from these regions^3,4^. The crossover frequency, at which $\mathrm{Re} \left[ \tilde{f}_{\mathrm{CM}} \right]=0$, defines the critical transition between these two kinetic regimes and dictates the resulting cellular trajectory.

Consequently, DEP force can be strategically utilized for cell separation based on divergence in either physical size or intrinsic dielectric properties. Since the magnitude of the force experienced by a cell is directly proportional to the cube of its radius (*a*^3^), cells of varying sizes exhibit markedly distinct kinetic responses when subjected to identical field conditions. Moreover, cells with disparate dielectric properties respond uniquely to specific excitation frequencies, experiencing forces of varying magnitudes or even opposite directions. In practical microfluidic applications, dielectric-based separation typically affords superior selectivity and resolution compared to purely size-based sorting. Accordingly, the μC-ASSIST platform leverages this dielectric contrast as the primary discriminating factor to achieve deterministic single-cell capture and high-purity isolation.

**Section S2. Acoustic-mediated cell membrane permeabilization**

The high viability and efficiency of biomolecular delivery achieved by the μC-ASSIST platform using megahertz surface acoustic waves (SAWs) represents a critical physical trade-off between minimizing cell damage and maximizing membrane permeability. This mechanism fundamentally differs from the inertial cavitation associated with conventional low-frequency ultrasound. Based on the Bilayer Sonophore (BLS) model, cell death and irreversible damage are governed by the maximal areal strain ($\varepsilon_{A,max}$) exerted on the cell membrane^5^. The BLS model treats the hydrophobic of the lipid bilayer as an absorbing interface where acoustic pressure induces tension and separation between the leaflets. Irreversible rupture occurs when this strain exceeds the membrane’s elastic limit. It also predicts the scaling of the $\varepsilon_{A,max}$ with the acoustic pressure amplitude $P_{A}$ and the excitation frequency $f_{\mathrm{SAW}}$:

$$\varepsilon_{A,max}\propto P_{A}^{0.8}f_{\mathrm{SAW}}^{-0.5}$$

(4)

This scaling law posits that lower frequencies elicit substantially greater membrane strains under a given acoustic pressure, thereby amplifying the probability of irreversible rupture. In contrast, the elevated frequencies utilized in the SAW method (MHz range) yield a decreased $\varepsilon_{A,max}$, consequently hindering inertial cavitation-like damage and promoting reversible membrane permeabilization.

While the BLS model explains the safety of high-frequency excitation, the effectiveness of SAW in inducing permeability is further explained by the direct coupling between surface acoustic waves and lipid bilayer structure, as demonstrated by Reusch et al.^6^ The transverse component of the SAW induces collective structural oscillation within the bilayer, characterized by periodic, reversible changes in membrane thickness ($\delta$) and electron density. The amplitude of this thickness oscillation is strongly dependent on the excitation frequency $f_{SAW}$, described by:

$$\delta\propto f_{\mathrm{SAW}}^{2}$$

(5)

This $f_{\mathrm{SAW}}^{2}$ dependence indicates that high-frequency SAW effectively transfers energy to the internal "soft modes" of the lipid structure. This process instigates localized, transient pore formation, thereby facilitating molecular transport without causing irreversible cellular damage.

Based on these theoretical principles, the μC-ASSIST platform achieves superior transfection performance by integrating deterministic cell immobilization with this acoustically mediated membrane permeabilization. In conventional bulk acoustofluidic systems, suspension cells experience acoustic radiation forces (ARF), leading to rapid cell translation or drifting away from the high-intensity acoustic zones^7^. This ARF-induced displacement severely attenuates the effective acoustic energy transferred to the cell membrane. In contrast, our device utilizes microcapillary-assisted DEP to rigidly capture the target cell at the microcapillary orifice *prior to* acoustic excitation. Notably, the near-field DEP force (~nN) is at least an order of magnitude greater than the combined acoustic displacing forces (~pN), including both ARF and streaming-induced drag, providing sufficient confinement against acoustic-induced displacement. This stable immobilization maintains the target cell within the localized acoustic field and facilitates effective interaction between the incident SAW and the cell membrane. According to the BLS and Reusch models, the mechanical stress generated by high-frequency SAW excitation induces membrane deformation and transient pore formation. Beyond its role in stabilizing cell position, the specific microcapillary geometry has been shown to further enhance acoustic–cell interaction by distorting the local acoustic field and generating strong boundary-driven acoustic streaming^8^. The phenomenon of localized streaming is understood to emerge from the interplay between high-frequency acoustic waves and solid microstructures. In this context, it functions as an active micro-pump, propelling exogenous cargos toward and through the transient pores formed in the membrane. Consequently, the synergy of single-cell capture and SAW-mediated membrane permeabilization enables the platform to achieve high transfection efficiency while preserving high cell viability.

**Supporting Figures**

**Fig. S1.** Schematic illustration of high-frequency SAW-mediated reversible sonoporation in a cell membrane, depicting four main stages: (a) membrane compression, (b) bilayer separation, (c) nanopore formation, and (d) pore resealing***.***

**Fig.** **S2.** **Characterization of the electric-field redistribution before and after single-cell trapping.** (a) Simulated ∇|*E*|²around the microcapillary opening without and with a captured cell. The red dashed lines indicate the sampling paths at *x* = 0 μm, extending from *y* = -10 to -35 μm, used for the quantitative analysis in (b). (b) Comparison of ∇|*E*|² along the axial centerline extending from the microcapillary opening into the main channel before and after cell capture. (c) Equivalent circuit model of the unloaded μC-ASSIST device. (d) Equivalent circuit model after single-cell trapping. The circuit elements represent the following physical components: R_h_ and R_l_ are the effective ohmic resistances of the main sample channel and side liquid electrode channel, respectively; C_PDMS_ and R_PDMS_ represent the capacitance and resistance of the insulating PDMS partition; R_μC_ and C_μC_ describe the intrinsic impedance of the electrolyte-filled microcapillary; R_gap_ and C_gap_ represent the leakage impedance associated with the residual gap between the trapped cell and microcapillary; C_mem_ and R_cyto_ correspond to the membrane capacitance and cytoplasmic resistance of the trapped cell.

**Fig. S3.** Impact of microcapillary dimensions on the maximum ∇|*E*|². (a) ∇|*E*|² as a function of microcapillary length (width fixed at 2 μm, frequency 100 kHz). (b) ∇|*E*|² as a function of microcapillary width (length fixed at 20 μm, frequency 100 kHz).

**Fig. S4.** Fabrication process flow of the μC-ASSIST chip.

**Fig. S5.** Computed real part of the effective CM factor (Re[$\tilde{f}_{cm}$]) for A549 cells (solid lines) and WBCs (dashed lines) as a function of the logarithmic frequency at different medium conductivities (10, 100, and 1000 μS/cm) by MATLAB 2020b. At a medium conductivity of 100 μS/cm (red lines), the A549 cells exhibit a crossover frequency at *f*_x011_ (~30 kHz), while WBCs exhibit a higher crossover frequency at *f*_x021_ (~120 kHz).

**Fig. S6.** Single-cell trapping efficiency. (a) Trapping efficiency vs. frequency at 45 V_rms_ and 1 mm/s. (b) Trapping efficiency vs. voltage at 120 kHz and 1 mm/s.

**Fig. S7.** Effect of SAW exposure duration and power on PI delivery efficiency and cell viability. (a) illustrates the PI delivery efficiency as a function of the SAW exposure time (5, 10, and 15 s) for various input radio frequency (RF) powers (0.4 to 0.8 W). (b) displays the percentage of cell viability after SAW treatment under the same exposure conditions as in (a).

**Fig. S8.** (a) SEM and (b) bright-field images of the scaled-up μC-ASSIST chip featuring an array of 15 microcapillary pairs. (c) Fluorescence image showing uniform single-cell capture across the array. Scale bars: 20 μm.

**Fig. S9.** Fluorescence microscopy images confirming RFP expression 48 h after SAW-mediated delivery of the mRFP-C3 plasmid. Left: Wide-field view (scale bar: 20 μm). Right: Magnified views (scale bars: 10 μm) of a representative single cell showing nuclear localization of the RFP signal.

**Fig. S10.** Intracellular delivery of Cy3‑labeled GAPDH siRNA. (a) Bright-field image of A549 cells. (b) Corresponding red fluorescence image showing Cy3-siRNA uptake. (c) Quantification of delivery efficiency. Scale bars: 50 μm.

**Fig. S11.** Comparison of cell migration rates at 24 and 48 h under control, DEP-only, and DEP-SAW groups.

**Supporting Tables**

**Table S1.** Comparison with the reported DEP devices in terms of the field strength and the gradient

| **DEP Method** | **Electrode/**  **structure** | **Excitation** | **Max field (V/m)** | **Max ∇\|*E*\|² (V^2^/m^3^)** | **Ref.** |
| --- | --- | --- | --- | --- | --- |
| Electrode-based DEP (eDEP) | Planar metal electrode | / | ~10^8^ | ~10^16^ | ^9^ |
| Contactless DEP  (cDEP) | Platinum  Electrodes with PDMS membrane | 40-200 kHz, 100-500 V_rms_ | / | ~10^12^ | ^10^ |
| Insulator-based DEP (iDEP) | Bipolar electrodes | 100 kHz, 30-60 Vpp | ~10^5^ | / | ^11^ |
| iDEP | Copper tape electrodes | 100 kHz-5MHz, 2-10 Vpp | / | 10^14^~10^15^ | ^12^ |
| Microcapillary-based DEP(μC-DEP) | Liquid electrode | 10 kHz-320kHz, 21.2V_rms_ | ~10^7^ | ~10^19^ | This work |

**Table S2.** Cell properties of A549 and WBC used for frequency-dependent $Re\left[ \tilde{f}_{\mathrm{CM}} \right]$ computation

| **Parameter** |  | **Unit** | **A549** | **WBC** | **Reference / Basis** |
| --- | --- | --- | --- | --- | --- |
| Radius ($R$) |  | μm | 7.5 | 3.75 | Experimentally measured |
| **Cytoplasm** | | |  |  |  |
| Electrical conductivity ($\sigma_{cyto}$) | | S/m | 0.23 | 0.6 | ^13^ |
| Relative permittivity ($\varepsilon_{cyto}$) | | / | 100 | 111 | ^14^ |
| **Cell membrane** | | |  |  |  |
| Electrical conductivity ($\sigma_{mem}$) | | S/m | 2🞨10^-6^ | 1🞨10^-6^ | ^13^ |
| Membrane thickness (d) | | nm | 5 | 5 |  |
| Membrane capacitance ($C_{mem}$) | | mF/m² | 10 | 5 | ^15-17^ |

**Note**: The membrane capacitance represents the effective specific membrane capacitance used in the single-shell model. The corresponding membrane relative permittivity was calculated from $\varepsilon_{mem}=\frac{C_{mem}d}{\varepsilon_{0}}$.

**Table S3:** Comparison with acoustic-mediated platforms

| **Delivery mechanism** | **Cell state and type** | **Selective single-cell capture** | **Treatment condition** | **Cargo type** | **Transfection efficiency** | **Cell viability** | **Ref.** |
| --- | --- | --- | --- | --- | --- | --- | --- |
| High-frequency SAW | Adherent (HEK293T, HeLa) | No | 10 MHz, 2 W, 0.5-10 min | siRNA (GAPDH) | / | >97% | ^18^ |
| Acoustothermal | Adherent (MCF-7, MSCs, CD3^+^ T cells) | No | 24 MHz, 10 V, 10 min | pDNA (EGFP, CXCR4, BDNF) | 80-90% | 85.9% (MCF-7);  83.9% (MSCs);  84.4% (CD3^+^ T cells) | ^19^ |
| SonoPIN | Adherent (4T1, MSCs) | Yes (Aptamer-targeted, bulk) | 1 MHz, 0.5 W/cm², 60 s | PROTAC | / | > 99% | ^20^ |
| Acoustofluidic capillary | Suspension (Jurkat, PBMCs, HSPCs) | No | 3.3 MHz, 40 Vpp, 0.5-10 min | pDNA (eGFP) | 62% (Jurkat);  15% (PBMCs); 20% (HSPCs) | > 80% | ^21^ |
| High-frequency SAW | Suspension (Jurkat, HuT 78) | No | 30 MHz, 1.2 W, 10 s | siRNA (GAPDH) | / | 91% | ^22^ |
| μC-ASSIST | Suspension (A549) | Yes (Label-free) | 50 MHz, 0.6 W, 10 s | pDNA (mRFP-C3) | 78.1% | 83.7% | This work |

**Table S4.** Sequences of siRNA duplexes, PCR primers, and probes for Bcl-xL and GAPDH

| **Sequence name** | **Sequence (5’->3’)** |
| --- | --- |
| **siRNA duplexes** | |
| siBcl-xL sense | GUUUAGUGAUGUGGAAGAGAATT |
| siBcl-xL antisense | UUCUCUUCCACAUCACUAAACTT |
| **PCR primers and probes** | |
| Bcl-xLForward | CCCCTTCTCTGCTCCACCAC |
| Bcl-xL Reverse | GCCAAGGGAACTGAGGTGTG |
| Bcl-xLProbe | 5-HEX//TCCTCCGTCCAGCCGCCATTGC//3-BHQ1 |
| GAPDH Forward | CTGAACGGGAAGCTCACTGG |
| GAPDH Reverse | TAGCCCAGGATCCCCTTGAG |
| GAPDH Probe | 5-FAM//CCGACGCCTGCTTCACCACCTTCT//3-BHQ1 |

**Reference**

1	Zhang, C., Khoshmanesh, K., Mitchell, A. & Kalantar-zadeh, K. Dielectrophoresis for manipulation of micro/nano particles in microfluidic systems. *Anal. Bioanal. Chem.* **396**, 401-420 (2009).

2	Chai, H. et al. Capillarity Enabled Large‐Array Liquid Metal Electrodes for Compact and High‐Throughput Dielectrophoretic Microfluidics. *Adv. Mater.* **36**, 2470154 (2024).

3	Luo, Y., Cao, X., Huang, P. & Yobas, L. Microcapillary-assisted dielectrophoresis for single-particle positioning. *Lab Chip* **12**, 4085-4092 (2012).

4	Tian, Z., Wang, X. & Chen, J. On-chip dielectrophoretic single-cell manipulation. *Microsyst. Nanoeng.* **10**, 117 (2024).

5	Krasovitski, B., Frenkel, V., Shoham, S. & Kimmel, E. Intramembrane cavitation as a unifying mechanism for ultrasound-induced bioeffects. *Proc. Natl. Acad. Sci. U. S. A.* **108**, 3258-3263 (2011).

6	Reusch, T. et al. Collective Lipid Bilayer Dynamics Excited by Surface Acoustic Waves. *Phys. Rev. Lett.* **113**, 118102 (2014).

7	Bruus, H. Acoustofluidics 7: The acoustic radiation force on small particles. *Lab Chip* **12**, 1014-1021 (2012).

8	Nama, N., Huang, P.-H., Huang, T. J. & Costanzo, F. Investigation of acoustic streaming patterns around oscillating sharp edges. *Lab Chip* **14**, 2824-2836 (2014).

9	Wang, Y. et al. A Two-Stage Separation of Circulating Tumor Cells Based on Deterministic Lateral Displacement and Dielectrophoresis Techniques. *IEEE Access* **9**, 143847-143859 (2021).

10	Shafiee, H., Caldwell, J. L., Sano, M. B. & Davalos, R. V. Contactless dielectrophoresis: a new technique for cell manipulation. *Biomedical Microdevices* **11**, 997 (2009).

11	Rathnaweera, T. N. & Anand, R. K. iDEP-based single-cell isolation in a two-dimensional array of chambers addressed by easy-to-align wireless electrodes. *Lab Chip* **25**, 1600-1610 (2025).

12	Islam, M. N., Jaiswal, B. & Gagnon, Z. R. High-Throughput Continuous Free-Flow Dielectrophoretic Trapping of Micron-Scale Particles and Cells in Paper Using Localized Nonuniform Pore-Scale-Generated Paper-Based Electric Field Gradients. *Anal. Chem.* **96**, 1084-1092 (2024).

13	Rahmati, M. & Chen, X. Separation of circulating tumor cells from blood using dielectrophoretic DLD manipulation. *Biomedical Microdevices* **23**, 49 (2021).

14	Uddin, M. R. & Chen, X. Enhancing cell separation in a hybrid spiral dielectrophoretic microchannel: Numerical insights and optimal operating conditions. *Biotechnology Progress* **40**, e3437 (2024).

15	Yang, J. et al. Dielectric Properties of Human Leukocyte Subpopulations Determined by Electrorotation as a Cell Separation Criterion. *Biophysical Journal* **76**, 3307-3314 (1999).

16	Han, S.-I., Joo, Y.-D. & Han, K.-H. An electrorotation technique for measuring the dielectric properties of cells with simultaneous use of negative quadrupolar dielectrophoresis and electrorotation. *Analyst* **138**, 1529-1537 (2013).

17	Gascoyne, P. R. C., Shim, S., Noshari, J., Becker, F. F. & Stemke-Hale, K. Correlations between the dielectric properties and exterior morphology of cells revealed by dielectrophoretic field-flow fractionation. *Electrophoresis* **34**, 1042-1050 (2013).

18	Ramesan, S., Rezk, A. R., Dekiwadia, C., Cortez-Jugo, C. & Yeo, L. Y. Acoustically-mediated intracellular delivery. *Nanoscale* **10**, 13165-13178 (2018).

19	Liu, X. et al. Acoustothermal transfection for cell therapy. *Sci. Adv.* **10**, eadk1855 (2024).

20	Wu, Y. et al. SonoPIN enables precise, noninvasive, and efficient intracellular delivery of PROTACs. *Proc. Natl. Acad. Sci. U. S. A.* **123**, e2534439123 (2026).

21	Belling, J. N. et al. Acoustofluidic sonoporation for gene delivery to human hematopoietic stem and progenitor cells. *Proc. Natl. Acad. Sci. U. S. A.* **117**, 10976-10982 (2020).

22	Ramesan, S. et al. Acoustofection: High-Frequency Vibrational Membrane Permeabilization for Intracellular siRNA Delivery into Nonadherent Cells. *ACS Applied Bio Materials* **4**, 2781-2789 (2021).
